# Predicting and exploring network components involved in pathogenesis in the malaria parasite via novel subnetwork alignments

**DOI:** 10.1186/1752-0509-9-S4-S1

**Published:** 2015-06-11

**Authors:** Hong Cai, Timothy G Lilburn, Changjin Hong, Jianying Gu, Rui Kuang, Yufeng Wang

**Affiliations:** 1 Department of Biology, University of Texas at San Antonio, San Antonio, TX 78249, USA; 2 Novozymes NA, Durham, NC 27709, USA; 3 Department of Computer Science and Engineering, University of Minnesota Twin Cities, Minneapolis, MN 55455, USA; 4 Department of Biology, College of Staten Island, City University of New York, Staten Island, NY 10314, USA; 5 South Texas Center for Emerging Infectious Diseases, University of Texas at San Antonio, San Antonio, TX 78249, USA

## Abstract

**Background:**

Malaria is a major health threat, affecting over 40% of the world's population. The latest report released by the World Health Organization estimated about 207 million cases of malaria infection, and about 627,000 deaths in 2012 alone. During the past decade, new therapeutic targets have been identified and are at various stages of characterization, thanks to the emerging omics-based technologies. However, the mechanism of malaria pathogenesis remains largely unknown. In this paper, we employ a novel neighborhood subnetwork alignment approach to identify network components that are potentially involved in pathogenesis.

**Results:**

Our module-based subnetwork alignment approach identified 24 functional homologs of pathogenesis-related proteins in the malaria parasite *P. falciparum*, using the protein-protein interaction networks in *Escherichia coli *as references. Eighteen out of these 24 proteins are associated with 418 other proteins that are related to DNA replication, transcriptional regulation, translation, signaling, metabolism, cell cycle regulation, as well as cytoadherence and entry to the host.

**Conclusions:**

The subnetwork alignments and subsequent protein-protein association network mining predicted a group of malarial proteins that may be involved in parasite development and parasite-host interaction, opening a new systems-level view of parasite pathogenesis and virulence.

## Background

Malaria is a severe global infectious disease. The World Health Organization estimated 207 million clinical cases, and 627,000 deaths due to malaria in 2012. Malaria infection was reported in 97 countries in 2013, representing a major public health concern. The vast majority of the deaths occurred in children under age 5 in sub-Saharan areas; it is estimated that a child dies from malaria every minute.

The causative agents of malaria are a group of parasites from the Genus *Plasmodium*. Among five human malaria parasites, *Plasmodium falciparum *causes the most severe form of malaria. It is notoriously difficult to study malaria biology as the parasite has a complex life cycle involving a mosquito vector and a human host. Within a human host, the parasite has a relatively dormant liver stage and an active red blood cell (RBC) stage. Clinical symptoms such as high fever, chills, headache, profuse sweats, fatigue, nausea, and vomiting are manifested at the blood stage, the stage that most drugs target against.

The effectiveness of antimalarial drugs, however, has been constantly challenged during the past decades due to the fast evolution of parasites that are resistant to multiple lines of drugs. This underscores an urgent need to search for novel drug targets. New lines of antimalarial targets have been identified [1-5] and are at various stages of functional and pharmacochemical characterization, thanks to the development of customized omic-based high throughput technologies, including genome sequencing and annotation [6-11], microarray [12-23], RNA-Seq [21,22,24], mass spectrometry [4,5,25-32], yeast two-hybrid protein-protein interaction assays [33-36], and large-scale compound screening [37]. Moreover, these amassing genomic, transcriptomic, proteomic, metabolomics [38-42], and interactomic data are enabling a new systems biology paradigm in malaria research; the complex and dynamic cellular processes such as pathogenesis and virulence have recently begun to be unveiled.

By nature, pathogenicity is an emergent property, that is, it is the result of the concerted effects of many genes. The suggestion by Mu et al. that differences in drug resistance in *P. falciparum *parasites from different continents were due to "overlapping, but not identical, sets of genes, including many encoding unknown proteins" [43] and the observation that the costs of maintaining antibiotic resistance in bacteria can be offset or lost thanks to changes at other loci not directly involved in antibiotic resistance [44] are both strongly suggestive of the reality of systems-level adaptation and of the existence of systems producing the virulence phenotype [45]. However, unlike bacterial systems, whose pathogenesis mechanism is much better characterized, to date, malaria pathogenesis, i.e., the development of the disease condition, remains largely unknown. A critical barrier is our limited knowledge of the cellular components, cellular events and reactions and other pathologic mechanisms associated with the disease development.

To unveil previously unknown proteins that are potentially involved in pathogenesis in *P. falciparum*, we developed a module-based subnetwork alignment approach. Traditional homology transfer using a protein-protein network alignment approach [46-54] does not appear feasible to predict functional othologs in the malaria parasite, because of the remote homology between *P. falciparum *and other known model organisms: BLAST-based sequence similarity search was unable to make functional annotation for over 60% of the genes in *P. falciparum *[11]. Our neighborhood subnetwork alignment algorithm [55] could potentially circumvent this limitation by searching for the similarities between functional modules. This algorithm, which was custom-developed for malaria research, was used to predicted novel transcriptional regulators and cell cycle regulatory proteins in *P. falciparum *[55,56]. In this paper, we extended the subnetwork alignment approach to identify the proteins related to pathogenesis and explore their potential functional roles.

## Results and discussion

### Twenty-four (24) novel proteins were predicted to be associated with pathogenesis in malaria parasite

Malaria pathogenesis is an emergent property that involves dynamic regulation of parasite metabolism, parasite signaling, and host-parasite interactions [57]. Gene Ontology using Term GO:0009405 (pathogenesis) predicted 95 *P. falciparum *genes (Additional File 1), not surprisingly, including 78 members of the *Plasmodium falciparum *erythrocyte membrane protein (PfEMP1) family. PfEMP1 is encoded by the highly polymorphic *Var *gene, and is one of the largest and most widely-studied protein families in *P. falciparum*. PfEMP1 is believed to play an important role in parasite pathogenesis and immune evasion [58]; it contributes to antigenic variation and cytoadherence, and the expression of specific PfEMP1 variants is associated with severe malaria in children [59].

Given the observed complexity of malaria pathogenesis, one might envisage that more network components have yet to be identified. Using a neighborhood subnetwork alignment algorithm, we predicted that 24 novel proteins in *P. falciparum *were functional orthologs of proteins known to be involved in pathogenesis in *E. coli *(Additional File 2). *E. coli *was chosen because its pathogenesis and virulence mechanisms are among the best-characterized of all known infectious agents. Seven of these predicted functional orthologs were annotated as "conserved *Plasmodium *proteins with unknown function", six of which do not have any predicted interactors in malarial protein-protein association networks (Table 1). Seventeen of these 24 predicted functional othologs may be involved in various biological processes from post-transcriptional regulation, protein translation, protein ubiquitination and modification, oxidation-reduction process, signaling, metabolism, to histone modification. Notably, one functional ortholog (PF3D7_0300100) is a variant of PfEMP1, and it is also consistent with the GO prediction that it may be associated with pathogenesis (Additional File 1).

**Table 1:** Novel *Plasmodium falciparum *proteins that were predicted to be functional orthologs of pathogenesis-related proteins in *E.coli*

PlasmoDB Accession Number	Annotation	Degree of Connectivity
PF3D7_1365900	60S ribosomal protein L40/UBI, putative	180

PF3D7_1241800	DEAD/DEAH box ATP-dependent RNA helicase, putative	98

PF3D7_0306800	T-complex protein beta subunit, putative	43

PF3D7_0823300	histone acetyltransferase GCN5 (GCN5)	20

PF3D7_1318700	conserved Plasmodium protein, unknown function	19

PF3D7_1212800	iron-sulfur subunit of succinate dehydrogenase	18

PF3D7_0208600	ribosome-recycling factor, putative (RRF)	16

PF3D7_1416900	prefoldin subunit 2, putative	15

PF3D7_0731100	Plasmodium exported protein (PHISTc), unknown function (GEXP11)	14

PF3D7_1013500	phosphoinositide-specific phospholipase C (PI-PLC)	11

PF3D7_0810600	RNA helicase, putative	9

PF3D7_1337200	1-deoxy-D-xylulose 5-phosphate synthase	7

PF3D7_1148800	Plasmodium exported protein (hyp11), unknown function	6

PF3D7_1008000	inositol polyphosphate kinase, putative (IPK1)	6

PF3D7_0919000	nucleosome assembly protein (NAPS)	6

PF3D7_1418000	ubiquitin fusion degradation protein UFD1, putative (UFD1)	5

PF3D7_0300100	erythrocyte membrane protein 1, PfEMP1 (VAR)	5

PF3D7_1342400	casein kinase II beta chain (CK2beta2)	1

PF3D7_1318700	conserved Plasmodium protein, unknown function	0

PF3D7_1364200	conserved Plasmodium protein, unknown function	0

PF3D7_1229300	conserved Plasmodium protein, unknown function	0

PF3D7_1451200	conserved Plasmodium protein, unknown function	0

PF3D7_0310900	conserved Plasmodium protein, unknown function	0

PF3D7_1008100	conserved Plasmodium protein, unknown function	0

### The set of functional orthologs is involved in important biological processes that are related to pathogenesis and virulence

**Figure 1:**
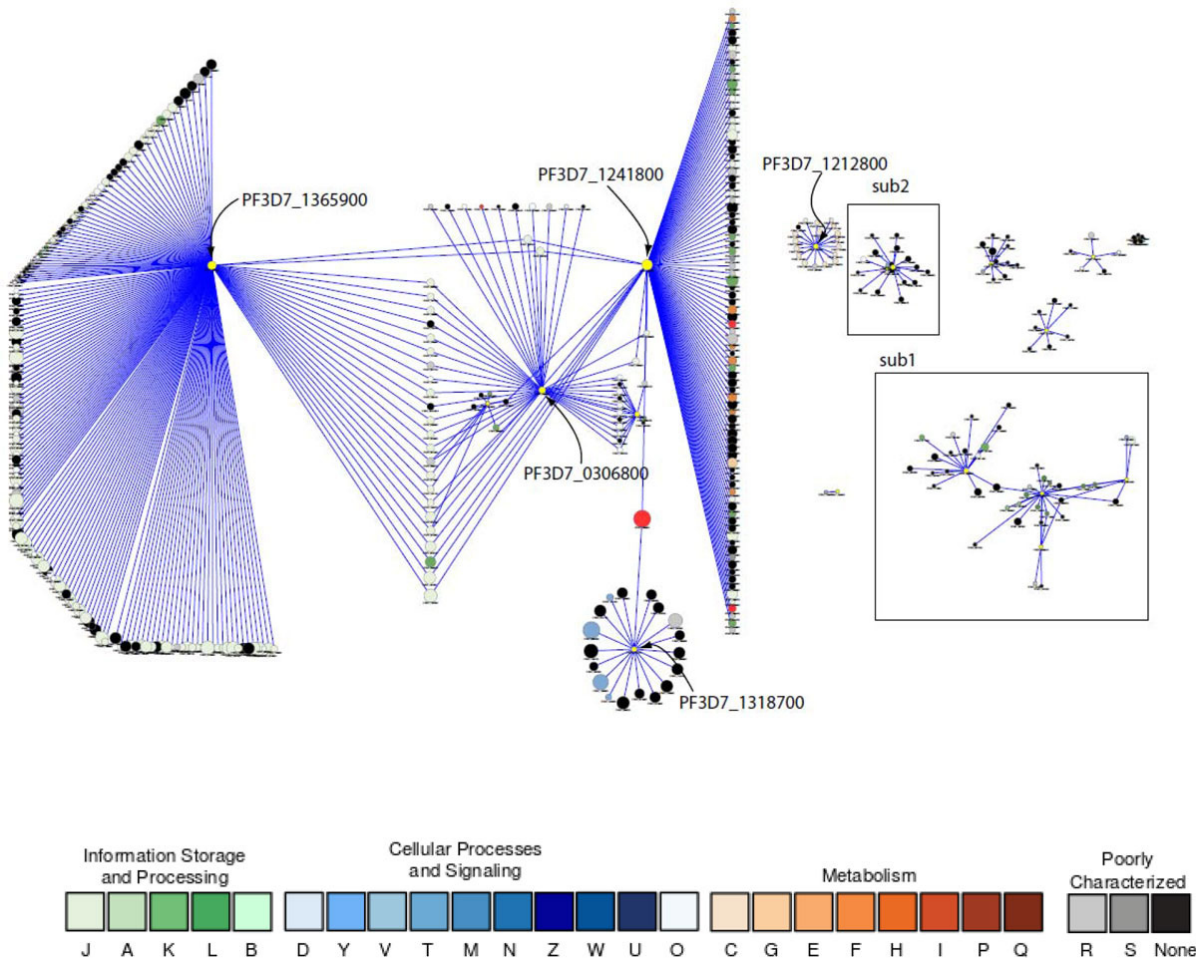
**Predicted protein-protein association networks related to pathogenesis in *P. falciparum***. Yellow nodes represent the predicted functional othologs of *E. coli *pathogenesis proteins. Node size is proportional to the degree of the connectivity of the node. Nodes are colored according to their functional classification in the eggNOG database [80]. The COG categories are [81] (J) Translation, ribosomal structure and biogenesis, (A) RNA processing and modification, (K) Transcription, (L) Replication, recombination and repair, (B) Chromatin structure and dynamics, (D) Cell cycle control, cell division, chromosome partitioning, (Y) Nuclear structure, (V) Defense mechanisms, (T) Signal transduction mechanisms, (M) Cell wall/membrane/envelope biogenesis, (N) Cell motility, (Z) Cytoskeleton, (W) Extracellular structures, (U) Intracellular trafficking, secretion, and vesicular transport, (O) Posttranslational modification, protein turnover, chaperones, (C) Energy production and conversion, (G) Carbohydrate transport and metabolism, (E) Amino acid transport and metabolism, (F) Nucleotide transport and metabolism, (H) Coenzyme transport and metabolism, (I) Lipid transport and metabolism, (P) Inorganic ion transport and metabolism, (Q) Secondary metabolites biosynthesis, transport and catabolism, (R) General function prediction only, and (S) Function unknown. Only the interactions among the nodes having confidence scores (S values from STRING [82,83]) greater than 0.7 were considered in this paper and are shown in the figure. The subnetworks labeled sub1 and sub2 appear in Figures 2 and 3 respectively.

We performed network mining on the 24 predicted pathogenesis-related functional orthologs. Eighteen proteins are associated with 418 other proteins with high confidence score (>0.7). The network centralization parameter, a measure of the distribution of network density [60], is 0.0548, indicating that the network is decentralized. The degree of connectivity of these 18 proteins ranges from 180 to one and the degree distribution offers a good fit to a power law distribution (Table 1). This type of distribution indicates that most nodes are connected to a few others and a few nodes are connected to many others (Figure 1). The network heterogeneity coefficient assesses the relative number of hubs in a network; in this network it is relatively high (1.455), which is in accord with the observed power law distribution.

Table 2 shows representative functional categories of the proteins that are associated with predicted pathogenesis-related *P. falciparum *proteins. They are active players in crucial cellular processes in parasite development, growth, and response to host and environmental stresses. In the following sections, we discuss critical network components and their potential associations with pathogenesis and virulence.

**Table 2:** Representative *P.falciparum *proteins that were predicted to be associated with pathogenesis.

Functional category	PlasmoDB Accession Number	Annotation
DNA replication	PF3D7_0111300	replication factor c protein, putative

Transcription and transcriptional regulation	PF3D7_0923000	DNA-directed RNA polymerase II, putative
	
	PF3D7_1206600	DNA-directed RNA polymerase III subunit, putative
	
	PF3D7_0206600	transcription factor, putative
	
	PF3D7_1451400	transcriptional regulatory protein sir2b (Sir2B)
	
	PF3D7_1342700	transcription activator, putative

Translation	PF3D7_1460700	60S ribosomal protein L27, putative
	
	PF3D7_1421200	40S ribosomal protein S25, putative
	
	PF3D7_1451100	elongation factor 2
	
	PF3D7_0422700	eukaryotic translation initiation factor 4F complex

Protein phosphorylation and signaling	PF3D7_0826700	receptor for activated C kinase homolog, PfRACK
	
	PF3D7_0424500	serine/threonine protein kinase, FIKK family (FIKK4.1)
	
	PF3D7_0623800	protein kinase, putative (TKL4)

Regulation of cell cycle	PF3D7_1428300	proliferation-associated protein 2g4, putative
	
	PF3D7_0619400	cell division cycle protein 48 homologue, putative

Proteolysis	PF3D7_0933600	mitochondrial-processing peptidase subunit beta, putative (MAS1)
	
	PF3D7_0904400	signal peptidase complex subunit 3, putative (SPC3)
	
	PF3D7_1331300	signal peptidase 21 kDa subunit (SP21)
	
	PF3D7_1130400	26S protease subunit regulatory subunit 6a, putative

Heat shock response	PF3D7_0708800	heat shock protein 110 (HSP110c)
	
	PF3D7_1357800	TCP-1/cpn60 chaperonin family, putative
	
	PF3D7_1215300	10 kd chaperonin, putative
	
	PF3D7_0308200	TCP-1/cpn60 chaperonin family, putative
	
	PF3D7_0214000	TCP-1/cpn60 chaperonin family, putative

1. Tight associations between translation, protein turnover, proteolysis, signaling, and stress response

The predicted functional ortholog with the highest connectivity (180) is a putative 60S ribosomal protein L40/UBI (PF3D7_1365900) (Figure 1). This protein is unique as it is a fusion protein of two domains: a C-terminal ribosomal L40e family domain and an N-terminal ubiquitin domain. It therefore plays dual functions: On one hand, it is a central member of the ribosomal protein complex in *P. falciparum*, which includes small and large subunit ribosomal proteins of varying sizes. This complex is associated with several translation initiation and elongation factors. On the other hand, L40/UBI is implicated in the ubiquitin-proteasome system (UPS) [61]. UPS is an essential mechanism in *P. falciparum *for degrading misfolded or unneeded proteins [62]. UPS is becoming a promising target for antimalarial development due to its critical roles in cell cycle regulation and stress response and the relative low toxicity of its inhibitors [63]. Notably, malarial L40/UBI may be involved in other processes that are currently not fully appreciated, such as (1) signaling. L40/UBI and PfRACK (*P. falciparum *receptor for activated C kinase homolog, PF3D7_0826700) are associated with a high confidence score (0.908), suggested by the co-expression of their homologs in *Caenorhabditis elegans, Drosophila melanogaster*, and humans, and protein-protein interaction found by affinity capture-MS assay in humans. The evidence that PfRACK could directly inhibit inositol 1,4,5-trisphosphate receptor-mediated calcium-signaling in mammalian host cells is indicative of a pathogenesis mechanism acting through the disruption of host activities [64]. (2) Genome stability. L40/UBI is associated with two putative proliferating cell nuclear antigens (PCNAs), auxiliary factors of DNA polymerase. Ubiquitin, along with SUMO (small ubiquitin-related modifier), were shown to effectively regulate DNA damage recognition and repair in yeast [65]. A similar mechanism, if it exists in the malaria parasite, would promote the accuracy of replication and timely repair, both of which are crucial for parasite survival within the host.

2. RNA metabolism and transcriptional regulation

The predicted pathogenesis-related functional orthologs with the second largest connectivity (98) is a putative DEAD/DEAH box ATP-dependent RNA helicase (PF3D7_1241800) (Figure 1). The main function of this family of RNA helicases is to unwind RNA [66]. Helicase is considered to be a potential antimalarial target due to its essentiality for parasite life cycle [67]. Fifty-one DEAD/DEAH helicase homologs were identified in *P. falciparum *by genomic analysis [68]. Our network mining indicated that PF3D7_1241800 is broadly involved in RNA metabolism; it is associated with (1) several RNA polymerase subunits and a number of other helicases required for transcription, (2) nucleolar GTP-binding proteins, putative GTPases, and BRIX-domain containing proteins for ribosomal biogenesis and maturation, (3) at least three putative U3 snoRNP associated proteins for pre-rRNA processing, (4) a ribosomal subunit export protein for RNA transport, and (5) an eukaryotic initiation factor required for translation. In addition to RNA metabolism, PF3D7_1241800 is associated with a putative transcription factor (PF3D7_0206600). This transcription factor is a member of the general transcription factor TFIIS family with a characteristic zinc ribbon conformation. The accurate information flow of basal transcriptional regulation and RNA metabolism is essential for parasite development.

3. Chromatin remodeling, epigenetic regulation, and antigenic variation

**Figure 2:**
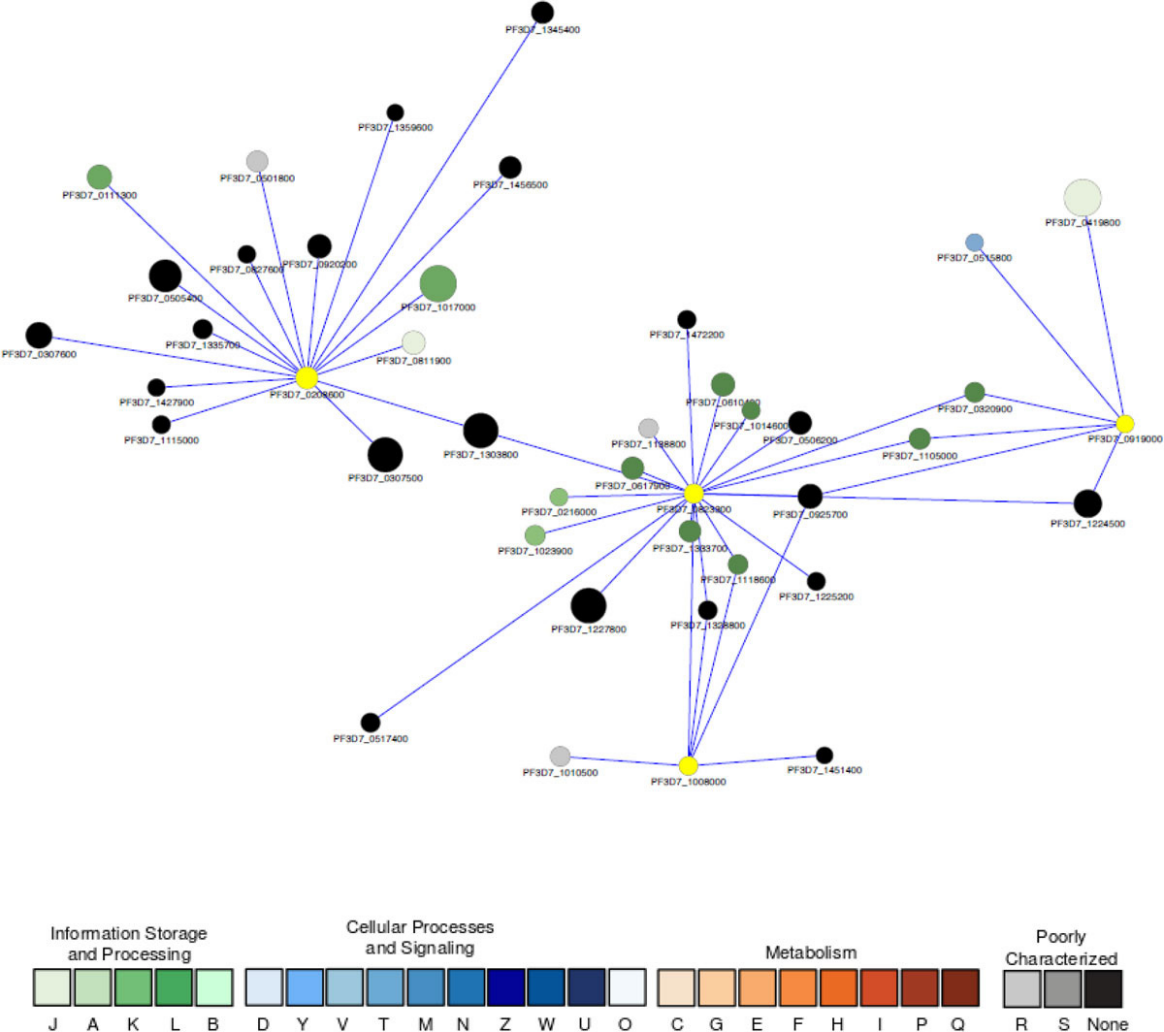
**A subnetwork showing the proteins associated with a histone acetyltransferase GCN5 (PF3D7_0823300)**. Node size is proportional to the degree of the connectivity of the node. Nodes are colored according to their functional classification in the eggNOG database [80]. The visualization is as for Figure 1.

It is becoming clear that chromatin remodeling/histone modification controls genome-wide gene expression in *P. falciparum *and epigenetic regulation may be a neglected major contributing factor to antigenic variation and evasion of the human immune system during parasite pathogenesis [69-71]. Our network analysis identified a subnetwork module centered on PfGCN5 (PF3D7_0823300), a bona fide histone acetyltransferase. PfGCN5 is associated with 20 other proteins (Figure 2), including histones H2A, H3, and H4, DNA helicase, three histone deacetylases, a putative chromodomain-helicase-DNA-binding protein, and a putative chromatin assembly protein ASF1, suggesting its role in chromatin remodeling. In addition, PfGCN5 may be involved in transcriptional regulation: it is associated with the transcriptional activator ADA2 with a confidence score of 0.955 [72]; in yeast, GCN5, ADA2, and ADA3 interact and form a catalytic core for histone acetyltransferase (HAT) activity. PfGCN may also have protein-protein interactions with a general transcription initiation factor TFIID and a putative transcriptional regulator. Interestingly, PfGCN5 is predicted to be associated with a transcriptional regulatory protein Sir2a (PF3D7_1328800) (Figure 2). Sir2a and its paralog Sir2b are implicated in epigenetic regulation of PfEMP1 antigenic variation, thus controlling the pathogenicity of malaria [73,74].

PfGCN5, is also indirectly associated with three predicted pathogenesis-related functional orthologs (labelled in yellow in Figure 2): (1) an NAPS protein (PF3D7_0919000) involved in nucleosome assembly, (2) a putative ribosome-recycling factor (RRF, PF3D7_0208600) that is essential for recycling of organellar ribosomes during protein translation, and (3) a putative inositol polyphosphate kinase (IPK1, PF3D7_1008000) that may be involved in signaling, chromatin remodeling, and mRNA export.

**Figure 3:**
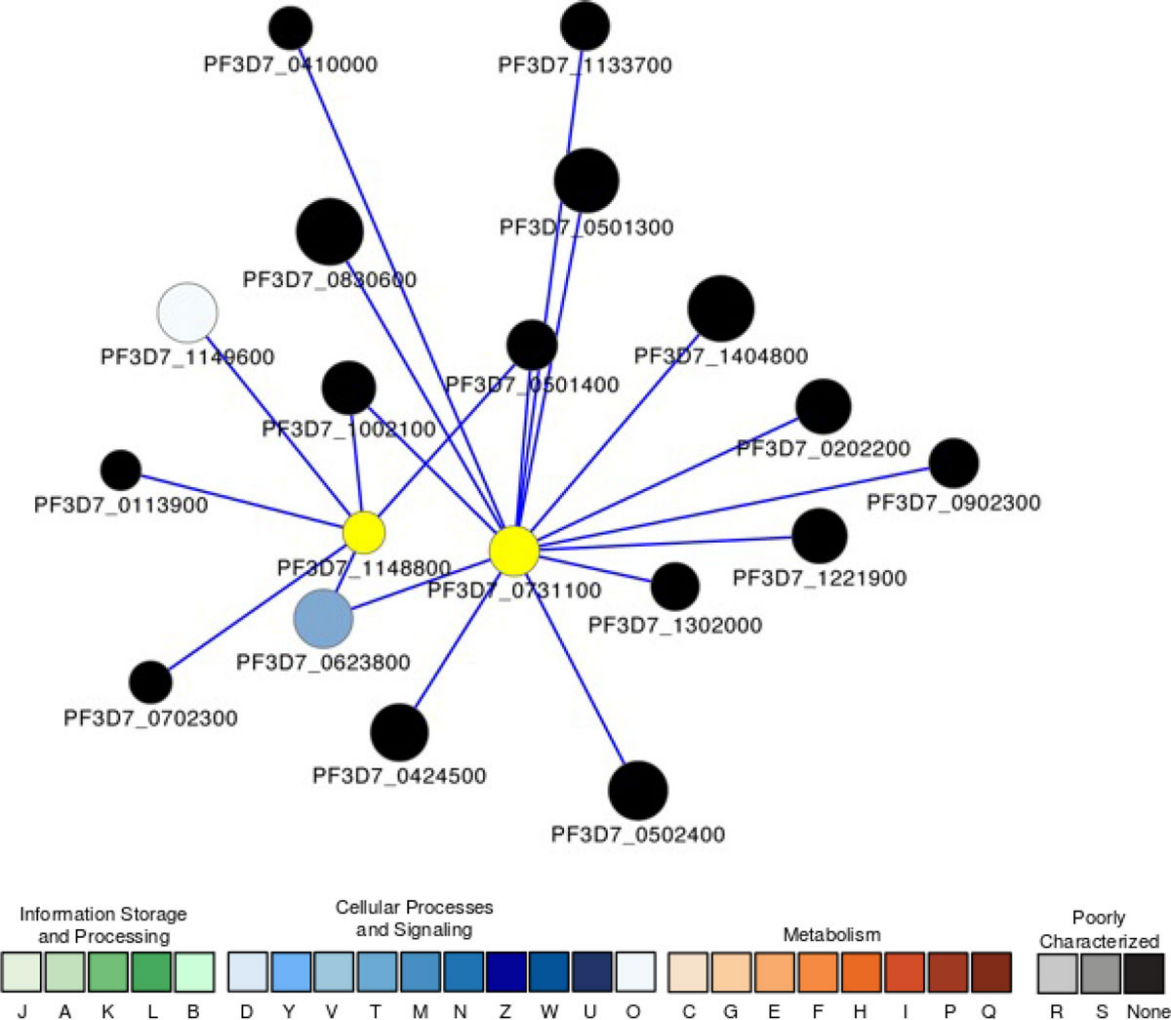
**A subnetwork showing the proteins associated with two putative *Plasmodium *exported proteins (PF3D7_0731100 and PF3D7_1148800)**. Node size is proportional to the degree of the connectivity of the node. Nodes are colored according to their functional classification in the eggNOG database [80]. The visualization is as for Figure 1.

Unlike the PfGCN5-centered chromatin-remodeling module, very little is known about another subnetwork centered on two *Plasmodium *exported proteins hyp11 (PF3D7_1148800) and GEXP11 (PF3D7_0731100) (Figure 3). The function of these two proteins is unclear, and the majority of their interactors are hypothetical proteins as well; the evidence of association is mainly established based on co-expression profiles and does not necessarily suggests direct functional association.

## Conclusions

Using a neighborhood subnetwork alignment algorithm, we identified network components associated with 24 potential pathogenesis-related proteins that may be involved in malaria pathogenesis. The proteins play roles in parasite information processing, signaling, epigenetic regulation, and entry to the host, all processes that are related to pathogenesis and virulence. A better understanding of the network components containing these proteins creates a new list of potential rational targets for antimalarial intervention.

## Methods

### Querying subnetworks using neighborhood alignments

We have framed the prediction of functional orthologs in *P. falciparum *as a subnetwork querying problem. Network alignment algorithms search the network of the organism of interest for subnetworks that are similar to subnetworks in other, better-understood organisms [75,76]. The network we used to search the "target" network is a well-studied functional module from a model organism. Such queries can find similar modules in the less studied target organism, permitting us to link information about biological functions across organisms [77]. We have used this method in previous studies to predict hitherto unrecognized transcriptional regulators and cell cycle regulators playing important roles in the parasite life cycle [55,56]. We adopted the same algorithm to identify proteins involved in pathogenesis.

We first mapped a set of proteins related to pathogenesis (GO: 0009405) from *E. coli *onto its own PPI network. A set of "neighbors" was selected for each pathogenesis protein in *E. coli*, creating a set of subnetworks, which, by inference, form a network of subnetworks in the query network. Similarly, each *P. falciparum *protein was mapped into its own PPI network, and a subnetwork of neighbors was constructed. Subnetwork size was controlled by including only proteins that are *k *hops from the central protein in each case and *k *was chosen such that the neighbor size was under 500, unless the central protein had more than 500 directly connected neighbors.

After deducing the neighborhood subnetworks for both the query *E. coli *pathogenesis-related proteins and all *P. falciparum *proteins, the *E. coli *subnetworks were combinatorically aligned against the *P. falciparum *subnetworks. When good alignments were found (see below), the central protein of the best-aligned *P. falciparum *subnetwork was labeled a functional ortholog of the cognate protein at the center of the relevant pathogenesis subnetwork in *E. coli*.

In order to analyze and score the degree of similarity between the *P. falciparum *and *E. coli *neighborhood subnetworks a shortest-path graph kernel was used to measure the similarity between two labeled networks, and a numerical score for each alignment was assigned [78]. To optimize the graph kernel, only paths between the central protein and other subnetwork proteins are counted. Each shortest path through the central protein can be considered a chain of cellular activities, and the path defines the dynamic function of this protein. Given a subnetwork *S_p _*with central protein *p *and a query subnetwork *S_q _*with central protein *q*, the shortest path similarity function is defined as follows,

$$K\left(Sq,Sp\right)=\frac{1}{\left|Sq|+|Sp\right|}\underset{\forall \left(i\;1,i2\right)\in Sq}{\prod }B\left(\left(i1,i2\right),Sp\right)$$

where

$$B\left(\left(i1,i2\right),Sp\right)=\underset{\forall \left(j1,j2\right)\in Sp}{\text{max}}\frac{2E\left(i1,j1\right)E\left(i2,j2\right)}{dist\left(i1,i2\right)+dist\left(j1,j2\right)},$$

$E\left(x,y\right)=exp\left(-\frac{Eval\left(x,y\right)}{\sigma }\right)$ with the normalization parameter σ = 10 measures the sequence similarity between proteins × and y based on the E-value of the sequence alignment, and *dist*(*x, y*) is the length of the shortest path connecting proteins × and y in the PPI subnetwork. The computation was done on a -log 10 scale. The method outlined here takes each pair of proteins (*i*1, *i*2) from one subnetwork and seeks the maximum ratio of sequence similarity with respect to the closeness (shortest path through the central protein) of the networks, in order to identify proteins (*j*1, *j*2) in the target subnetwork. Using this algorithm, a subnetwork alignment score is obtained by collecting the shortest paths between two neighborhood subnetworks, getting an alignment score for each pair of proteins, and totaling all of the alignment values. Hence, by quantifying the sequence similarity and network similarity and evaluating the role of the central protein in the query network we can summarize the functional coherence, and distance between two central proteins, as a numerical score.

### Network data and network analysis

We downloaded protein-protein interaction data for *E. coli *from the IntAct database [79], and protein-protein association data for *P. falciparum *from the STRING database [45]. STRING uses numerous data types, including sequence similarity estimates, pathway analysis, chromosome synteny, genome organization, and phylogenetic reconstruction, as well as literature text mining to estimate association confidence scores (S), ranging from 0.15 to 0.999. The data are integrated using a Bayesian approach and the scores approximate the likelihood of finding the pairs of proteins in the same pathway. Cytoscape 3.1 was used for network visualization [59]. EggNOG database was used for functional classification of the network nodes [48]. NetworkAnalyzer was used to compute topological parameters and centrality measures of the cellular networks [49].

## List of abbreviations used

GO: Gene Ontology

HAT: histone acetyltransferase

IPK: inositol polyphosphate kinase

PCNA: proliferating cell nuclear antigen

PfEMP1: *Plasmodium falciparum *erythrocyte membrane protein

PfRACK: *P. falciparum *receptor for activated C kinase

PPI: protein-protein interaction

RBC: red blood cell

RRF: ribosome-recycling factor

SUMO: small ubiquitin-related modifier

UPS: ubiquitin-proteasome system

## Competing interests

The authors declare that they have no competing interests.

## Authors' contributions

YW and RK conceived and designed the study. All authors performed bioinformatics data analysis and drafted the manuscript. All authors read and approved the final manuscript.

## Supplementary Material

Additional file 1 *P. falciparum *proteins that are annotated to be associate with Gene Ontology (GO) term GO0009405 (pathogenesis). Click here for file

Additional File 2 Predicted functional orthologs involved in pathogenesis in *P. falciparum*. The query genome is *P. falciparum*, and the target genome is *E. coli*. GO: Gene Ontology. BP: Biological Process. MF: Molecular Function. CC: Cellular Component. Click here for file

## Declarations

The publication costs for this article were funded by the corresponding author YW.

This article has been published as part of *BMC Systems Biology *Volume 9 Supplement 4, 2015: Selected articles from The International Conference on Intelligent Biology and Medicine (ICIBM) 2014: Systems Biology. The full contents of the supplement are available online at http://www.biomedcentral.com/bmcsystbiol/supplements/9/S4.
